# Supplementary material for: Reconciling Mining with the Conservation of Cave Biodiversity: A Quantitative Baseline to Help Establish Conservation Priorities
Source: PLoS One. 2016 Dec 20;11(12):e0168348. doi: 10.1371/journal.pone.0168348 (PMC5173368; doi:10.1371/journal.pone.0168348)
Supplement: S1 Dataset — (ZIP) [file pone.0168348.s002.zip › Taxa/Serra Norte/SN_2013/PES/Lista PESE-02.pdf]

CAVIDADE PESE-0002

Classe	Ordem	Fam/Outros	Gên/Outros	Espécie	S-U	U-S	Zon.
Annelida	Oligochaeta			sp.	8	3	P/A
Arachnida	Acari	(Oribatida)		sp.3	X		A
Arachnida	Acari	Laelapidae	*Cosmolaelaps*	sp.1		X	A
Arachnida	Acari	Laelapidae		sp.1	X		A
Arachnida	Acari	Macrochelidae		sp.1	X		A
Arachnida	Acari	Macronyssidae		sp.1		X	P
Arachnida	Acari	Opilioacaridae	*Neoacarus*	sp.1		X	A
Arachnida	Acari	Sarcoptiformes		sp.1		X	A
Arachnida	Acari	Trombiculidae		sp.2	X		E
Arachnida	Acari	Veigaiidae		sp.1		X	A
Arachnida	Amblypygi	Phrynidae	*Heterophrynus*	*longicornis*	12	14	P/A
Arachnida	Araneae	Corinnidae		jovem	1	3	A
Arachnida	Araneae	Ctenidae		jovem	1		E
Arachnida	Araneae	Pholcidae	*Mesabolivar*	*aurantiacus*	X		P
Arachnida	Araneae	Pisauridae	*Architis*	sp.1	4	2	E/P/A
Arachnida	Araneae	Theridiosomatidae	*Plato*	sp.1	X	X	P
Arachnida	Opiliones	Sclerosomatidae	*Prionostema*	sp.1	X	X	E
Arachnida	Opiliones	Stygnidae	*Protimesius*	*gracilis*	1		E
Arachnida	Pseudoscorpiones	Bochicidae		sp.1	X		A
Arachnida	Pseudoscorpiones	Chernetidae	*Spelaeochernes*	sp.1	X	X	P
Arachnida	Pseudoscorpiones	Chthoniidae	*Pseudochthonius*	sp.1	X	X	E
Diplopoda	Polydesmida	Fuhrmannodesmidae		sp.1	X	X	P/A
Diplopoda	Polydesmida	Fuhrmannodesmidae		sp.2		X	A
Diplopoda	Polydesmida	Pyrgodesmidae		sp.1	X	X	P/A
Diplopoda	Polydesmida	Pyrgodesmidae		sp.2	X		E/A
Entognatha	Collembola	Cyphoderidae		sp.1		X	P/A
Entognatha	Collembola	Entomobryidae		sp.1	X		E
Entognatha	Collembola	Paronellidae		sp.1	X	X	E/P/A
Entognatha	Collembola	Paronellidae		sp.4	X		A
Insecta	Coleoptera	Carabidae		sp.4	X		E
Insecta	Coleoptera	Elateridae		jovem	X		E
Insecta	Coleoptera	Staphylinidae	Pselaphinae	sp.2		X	P
Insecta	Coleoptera	Tenebrionidae		jovem	X		A
Insecta	Diptera	Cecidomyiidae	Cecidomyiinae	sp.	X	X	E
Insecta	Diptera	Chaoboridae		sp.	X		P
Insecta	Diptera	Culicidae		jovem	X		P
Insecta	Diptera	Drosophilidae	*Drosophila*	*eleonorae*	X		E
Insecta	Diptera	Psychodidae	*Pericoma*	sp.	X		E
Insecta	Diptera	Psychodidae	*Sciopemyia*	*sordellii*	X		P
Insecta	Diptera	Sciaridae		sp.		X	E
Insecta	Diptera	Tipulidae	Tipulinae	sp.	X		E/P/A
Insecta	Heteroptera	Cydnidae	*Pangaeus*	sp.1	X	X	P/A
Insecta	Homoptera	Cixiidae		sp.4		X	E
Insecta	Hymenoptera	Chalcidoidea		sp.3		X	E
Insecta	Hymenoptera	Diapriidae		sp.1	X		A
Insecta	Hymenoptera	Formicidae	*Acromyrmex*	sp.1	X		P
Insecta	Hymenoptera	Formicidae	*Acropyga*	sp.1	X		E
Insecta	Hymenoptera	Formicidae	*Camponotus*	sp.1		X	P
Insecta	Hymenoptera	Formicidae	*Crematogaster*	sp.1	X		E

Insecta	Hymenoptera	Formicidae	*Gnamptogenys*	sp.2	X	X	E/P
Insecta	Hymenoptera	Formicidae	*Nylanderia*	sp.1	X		E/P
Insecta	Hymenoptera	Formicidae	*Pachycondyla*	*striata*	1	1	E
Insecta	Hymenoptera	Formicidae	*Pheidole*	sp.2	X	X	E/P
Insecta	Hymenoptera	Formicidae	*Solenopsis*	sp.1	X		P/A
Insecta	Hymenoptera	Formicidae	*Solenopsis*	sp.2		X	P
Insecta	Orthoptera	Phalangopsidae	*Phalangopsis*	sp.	480	200	P/A
Gastropoda	Pulmonata	Bulimulidae	*Naesiotus*	sp.	X		A
Gastropoda	Pulmonata	Subulinidae	*Lamellaxis*	sp.	X	X	A
Gastropoda	Pulmonata	Systrophiidae	*Happia*	sp.	X	X	P/A
Malacostraca	Isopoda	Philosciidae		sp.1	X	X	A
Malacostraca	Isopoda	Scleropactidae		sp.1	X	X	A
Symphyla		Scutigerellidae	*Hanseniella*	sp.1	X	X	P/A
Amphibia	Anura	Leptodactylidae	*Leptodactylus*	cf. *vastus*		2	P
Mammalia	Chiroptera	Phyllostomidae	*Carollia*	*perspicillata*	10	3	A
Mammalia	Chiroptera	Phyllostomidae	*Phyllostomus*	sp.		15	A
